# The need for a re-evaluation of best supportive care studies reported to date

**DOI:** 10.1038/sj.bjc.6606081

**Published:** 2011-02-01

**Authors:** D C Currow, K Foley, S Y Zafar, J L Wheeler, A P Abernethy

**Affiliations:** 1 Discipline of Palliative and Supportive Services, Flinders University, Adelaide, South Australia, Australia; 2 Pain and Palliative Care Service, Memorial Sloan-Kettering Cancer Center, New York, USA; 3 Division of Medical Oncology, Department of Medicine, Duke University Medical Centre, Durham, NC, USA

The landmark study by Temel *et al*, of early referral to palliative care (i.e., best supportive care (BSC)) plus standard oncology care *vs* standard oncology care alone, gained widespread attention for its demonstration of improved survival and quality of life among lung cancer patients who received palliative care in addition to standard oncology care (Temel *et al*, 2010). As the study was not powered for survival as the primary outcome, these results—though undeniably exciting—require confirmation in subsequent studies. The findings are not likely to represent a type I error, given that this experimental design supports earlier observational population-based studies (Connor *et al*, 2007).

Equally important, though less touted than survival and quality-of-life impact, is this study's design, which bears operational, funding, and practice implications for all cancer clinical trials using BSC as a control arm (Zafar *et al*, 2008; Cherny *et al*, 2009; Temel *et al*, 2010). For years, many cancer clinical trials have been designed to evaluate the benefits of novel anti-cancer drugs against BSC; many drugs have been registered based on modest survival advantages when compared with BSC. Universally across these studies, however, BSC control arms have not been standardised in clinical trials; are not consistent with contemporary palliative care practice; are not based on the extensive best available evidence (Tieman *et al*, 2008); and are not described in sufficient detail to reproduce in subsequent studies. In research reported before the Temel paper in the *New England Journal of Medicine*, BSC was a flimsy label rather than denoting a reliable, consistently applied, replicable, or meaningful palliative care comparator for anti-cancer interventions.

What happens when clinical trials do not standardise BSC based on widely published, evidence-based supportive and palliative care, or when they do not carefully delineate what constitutes their BSC arm? The clinical care provided in the BSC arms of cancer clinical trials is haphazard; study results are difficult to interpret or misleading at worst and hard to generalise at best. For example, across studies that include a BSC arm, participants do not receive consistent symptom and needs assessment, nor consistent, ongoing, biopsychosocial nor clinical support. Results from one study, then, cannot be compared with another and, without a standardised backdrop for comparison, clinicians cannot know what to expect as results for a particular patient. The issues raised by differences in BSC across studies are magnified in multi-site randomised trials, where the composition of BSC practice is left to local discretion and practice. This heterogeneity of control arms stands in sharp contrast to the usual rigour of intervention arms, and indeed to all other aspects of clinical trials, wherein design elements such as chemotherapy dose, cycle, and dose modification are carefully standardised between collaborating sites, reported in publications, and evidence based.

Models to codify best supportive and palliative care are available for use in designing clinical trials, implementing studies across sites, and reporting results (Currow *et al*, 2009). Nonetheless, descriptions of BSC arms presented in the medical literature remain consistently poor (Zafar *et al*, 2008; Cherny *et al*, 2009).

Temel's study utilises an evidence-based definition of palliative care, mandates documentation, and thereby demonstrates the reproducible benefits of evidence-based BSC. Because of this transparency, results can be translated into clinical practice. By showing a survival advantage when early palliative care—carefully standardised and documented—accompanies standard oncology care, the findings of Temel *et al* immediately challenge the survival impact claimed by cancer trials that incorporate non-standardised BSC as a surrogate for evidence-based supportive and palliative care. The quality of those previous studies pales in comparison with that of the Temel trial, and their results are, correspondingly, questionable. In describing a BSC arm, implementing it as a standardised intervention, and conducting seminal comparative effectiveness research on its introduction into clinical care, Temel *et al* have set a new benchmark for BSC—a groundbreaking contribution.

Recognition of this advance should lead to a profound change. With a new standard for research design in cancer clinical trials, institutional review boards (IRBs, Ethics Committees), the Food and Drug Administration (FDA and the equivalent agencies internationally), research funders, and journal editors should no longer accept studies that compare interventions with ‘BSC’ that is non-standardised, inconsistent with contemporary practice, not evidence based, nor poorly described.

The paper by Temel *et al* highlights the poor quality of the control arm of many published studies. Given this concern, it is of immediate importance to patients and funders that rigorous pharmacovigilance studies be conducted to ensure that the marginal survival advantages demonstrated in studies of many recently registered anti-neoplastic therapies where BSC has been the control arm are being widely reproduced in clinical practice. When clinical decisions in real-world settings are based on trials in which half of all participants received suboptimal care (BSC) in comparison with the BSC benchmark set by Temel *et al*, patients may not realise the net clinical benefit, including the survival advantage that studies suggest.
